# Enhancing coherence via tuning coupling range in nonlocally coupled Stuart–Landau oscillators

**DOI:** 10.1038/s41598-018-27020-0

**Published:** 2018-06-07

**Authors:** Nannan Zhao, Zhongkui Sun, Wei Xu

**Affiliations:** Department of Applied Mathematics, Northwestern Polytechnical University, Xi’an, 710129 P.R. China

## Abstract

Nonlocal coupling, as an important connection topology among nonlinear oscillators, has attracted increasing attention recently with the research boom of chimera states. So far, most previous investigations have focused on nonlocally coupled systems interacted via similar variables. In this work, we report the evolutions of dynamical behaviors in the nonlocally coupled Stuart–Landau oscillators by applying conjugate variables feedback. Through rigorous analysis, we find that the oscillation death (OD) can convert into the amplitude death (AD) via the cluster state with the increasing of coupling range, making the AD regions to be expanded infinitely along two directions of both the natural frequency and the coupling strength. Moreover, the limit cycle oscillation (OS) region and the mixed region of OD and OS will turn to anti-synchronization state through amplitude-mediated chimera. Therefore, the procedure from local coupling to nonlocal one implies indeed the continuous enhancement of coherence among neighboring oscillators in coupled systems.

## Introduction

The investigation of coupled nonlinear oscillators model creates a favorable and effective platform for exploring various oscillatory patterns in physics, chemistry and neuroscience. Owing to different coupling scenarios among interacting oscillators with intrinsic properties, a host of novel collective phenomena can be introduced, such as synchronization^1–3^, chimera states^4,5^, and oscillation suppression^6,7^. Recent studies have demonstrated that the oscillation suppression can be separated into two types according to distinct generation mechanisms and manifestations^7,8^: (i) amplitude death (AD), where the oscillatory behavior of coupled oscillators disappears to produce a homogeneous steady state (HSS); (ii) oscillation death (OD), where the symmetry of coupled system is broken, and the inhomogeneous steady state (IHSS) occurs. In recent years, the researchers from different fields have proposed a variety of approaches to induce AD or OD, such as parameter mismatch^9^, various characteristics of propagation delay^10–12^, dynamical coupling^13^, conjugate (dissimilar) coupling^14–16^, mean-field diffusion coupling^17^ and nonlinear coupling^18^. In addition, the two oscillation quenching behaviors have been the subject of potential applications, for example, AD can be applied to adjust the output in laser systems^19^ and electronic circuits^20^, while OD is mainly used as the background mechanisms in cellular differentiation^21^ and synthetic genetic network^22^. Even so, AD and OD are not independent or opposite, and AD can transit to OD under certain coupling topologies^7,8,23,24^. In this paper, we also describe the mutual transitions between AD and OD in the nonlocally coupled high-dimensional system.

Numerous studies have indicated that coupling strategies play a significant role to determine the collective behaviors of coupled nonlinear systems. Initially, local coupling and global coupling, as two fundamental connection methods, have already received far-ranging concern, whereas the discussion of nonlocal coupling has not obtained as much attention. In 2002, Kuramoto *et al*.^25^ observed that an extraordinary class of oscillation patterns will appear if the coupling is nonlocal, in which the synchronization and desynchronization are concomitant in two different domains. Subsequently, Abrams and Strogatz^4^ called the novel phenomenon as chimera state to represent the coexistence of coherent oscillations and incoherent ones. Thenceforth, nonlocal coupling topology has often been considered as a necessary condition to induce chimera states, and different types of chimera states have been discovered in nonlocally coupled models, such as, amplitude-mediated chimera^26,27^, and pure amplitude chimera and chimera death^28,29^. Very recently, chimera states have also been found in global coupling^30^ and local coupling^31^, and the experimental observations have been offered in chemical^32^, electronic^33^, optical combs systems^34^. However, in the researches of chimera states, the interaction between the oscillators always utilizes the similar variables, and the effects of coupling via conjugate (or dissimilar) variables on chimera states have not been provided.

Practically, conjugate coupling (or dissimilar coupling) is popular and natural coupling manner in many real circumstances^35,36^. Recent studies have suggested that the conjugate coupling is more practical method than classical diffusive coupling in certain situations. For example, it can adjust effectively the emitting of light signal in the coupled semiconductor laser system^37^. Not only that, conjugate coupling can realize oscillation suppression of coupled identical oscillators in the absence of time delay^14^. However, most existing studies have focused mainly on two conjugate coupled oscillators, where the coupled systems are low-dimensional. Only recently, the role of conjugate coupling in locally coupled chaotic systems has been reported that stability region of synchronization is irrelevant to the number of oscillators^38^, and they also have observed the oscillation quenching and multistability phenomenon in locally conjugate coupled Stuart–Landau oscillators^16^. Compared with the local coupling, the relationship among oscillators becomes closer in the case of nonlocal coupling with the increasing of coupling range, but the collective dynamic behaviors of nonlocally coupled system still are not clearly described. Therefore, the main work of this paper is to investigate the changes of oscillation patterns in the transition from local coupling to nonlocal coupling, to further exploit the crucial role of coupling range.

Motived by the above analysis, we introduce the nonlocal coupling topology to Stuart–Landau oscillators with conjugate variables throughout this paper. In next section, based on the local coupling scenario where AD, OD and limit cycle oscillation (OS) can be observed in the phase diagram^16^, we try to reveal successively the variations of dynamic behaviors through tuning the coupling range from local coupling to nonlocal coupling. Through theoretical analysis, we can obtain the conditions for AD in different coupling ranges. Besides, the numerical results show that OD can transit to AD for the strong coupling, where the stable fixed points gather gradually to form cluster states and finally turn to a stable fixed point (i.e. AD) with the increase of coupling range. Moreover, we also find numerically for the weak coupling that OD and OS can convert to anti-synchronization via the amplitude-mediated chimera. Thus, this peculiar performance, in fact, portends the enhancement of coherence. Finally, the conclusions will be given.

## Results

Now, let us consider a ring of *N* identical nonlocally coupled Stuart-Landau oscillators, and the oscillators are coupled mainly through conjugate variables, the dynamic equation is shown as

1 $$\begin{array}{rcl}\frac{d{x}_{i}}{dt} & = & (1-{x}_{i}^{2}-{y}_{i}^{2}){x}_{i}-w{y}_{i}+\frac{\varepsilon }{2p}\sum _{k=i-p}^{i+p}({y}_{k}-{x}_{i}),\\ \frac{d{y}_{i}}{dt} & = & (1-{x}_{i}^{2}-{y}_{i}^{2}){y}_{i}+w{x}_{i}+\frac{\varepsilon }{2p}\sum _{k=i-p}^{i+p}({x}_{k}-{y}_{i}).\end{array}$$

where *i* = 1, 2, …, *N*, the parameter *ε* denotes the coupling strength, and *w* is the inherent frequency of the oscillators. For single uncoupled oscillator, it exhibits limit cycle oscillation with radius 1 and frequency *w*. Here *p* governs the number of nearest neighbors in each direction of the ring, and it is called the coupling range. Then *p* = 1 corresponds to the local coupling, and *p* = *N*/2 (*N* is even) or *p* = (*N* − 1)/2 (*N* is odd) is the global coupling. Thus, changing the value of *p* can establish the transition from the local coupling to the global coupling. Here the periodic boundary condition is implemented for the above system. In addition, the networks of *N* (*N* = 100) nonlocally coupled oscillators (Eq. (1)) are solved numerically by the fourth order Runge-Kutta method with integration step size *h* = 0.01, and the initial condition is adopted as follows: the former 50 oscillators are specified to start from positive constants (*x*_*i*_ = 0.3, *y*_*i*_ = 0.5, *i* = 1, … 50), and the other 50 oscillators select the opposite numbers (*x*_*i*_ = −0.3, *y*_*i*_ = −0.5, *i* = 51, … 100).

Obviously, the origin is a fixed point of the coupled system, and when it is stable, AD can appear. Hence, in the following we consider firstly the generation condition of AD through executing linear stability analysis method, and the Jacobian matrix *J* at the origin can be obtained as follows:

2 $$J={|\begin{array}{cccccc}A & \mathop{\overbrace{\begin{array}{ccc}A & \cdots  & B\end{array}}}\limits^{p} & O & \cdots  & O & \mathop{\overbrace{\begin{array}{ccc}B & \cdots  & B\end{array}}}\limits^{p}\\ B & \begin{array}{ccc}A & B & \cdots \end{array} & B & \begin{array}{cc}O & \cdots \end{array} & O & \begin{array}{ccc}B & \cdots  & B\end{array}\\ \vdots  & \ddots  &  & \ddots  &  & \vdots \\ B & \begin{array}{ccc}\cdots  & B & O\end{array} & \cdots  & O & B & \begin{array}{ccc}\cdots  & B & A\end{array}\end{array}|}_{N\times N}.$$

Here, the diagonal elements of the matrix *J* are *A*, and the left and right sides of the matrix *A* have *p* matrix *B* respectively according to the periodic boundary. Also *A* and *B* are matrix blocks of *R*_2×2_, namely

3 $$A=[\begin{array}{cc}1-\varepsilon  & -w\\ w & 1-\varepsilon \end{array}],\,B=[\begin{array}{cc}0 & \varepsilon /(2p)\\ \varepsilon /(2p) & 0\end{array}].$$

And the remaining elements are the zero matrix (*O*) of *R*_2×2_. Then, the characteristic equation to estimate AD of the coupled system can be derived by utilizing the property of the cyclic matrix^39^,

4 $${(1-\varepsilon -\lambda )}^{2}+{w}^{2}-(\frac{{\varepsilon }^{2}{\lambda }_{k}}{2p})=0,$$

where

5 $${\lambda }_{k}=\frac{\cos (\frac{2k\pi p}{N})-\,\cos (\frac{2k\pi (p+1)}{N})}{1-\,\cos (\frac{2k\pi }{N})}-1,$$

and *k* = 0, 1, …, *N* − 1. Through simple algebraic manipulations, one can further arrive at the characteristic value

6 $$\lambda =(1-\varepsilon )\pm \sqrt{{(\frac{\varepsilon {\lambda }_{k}}{2p})}^{2}-{w}^{2}}.$$

AD emerges if and only if all eigenvalues have negative real parts, so the critical conditions induced AD are given by

7 $$\,\,\{\begin{array}{l}\varepsilon  > 1,\,\\ \,[1-{(\frac{{\lambda }_{k}}{2p})}^{2}]{\varepsilon }^{2}-2\varepsilon +1+{w}^{2} > 0.\end{array}$$

**Figure 1:**
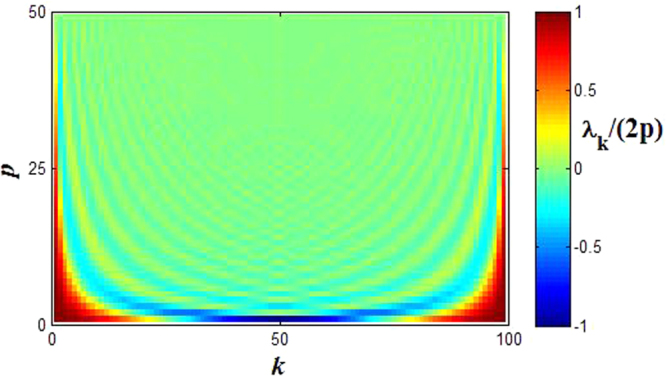
The change range of *λ*_*k*_/(2*p*) with the change of *k* and *p*, here *N* = 100.

**Figure 2:**
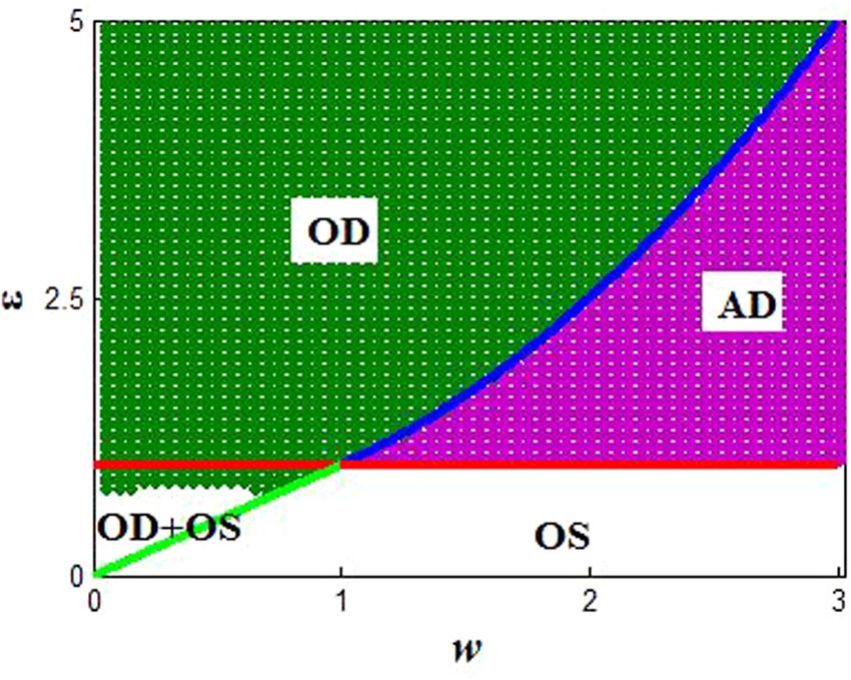
The phase diagram of coupled system (1) for local coupling (*p* = 1). Green region indicates oscillation death (OD) and purple region is amplitude death (AD), and the white region is limit cycle oscillation (OS), which is obtained by numerical simulations. The red, blue and green lines are the boundaries based on the theoretical results. Here the parameter *N* = 100.

It should note that the lower bound of coupling strength to induce AD is *ε* = 1, and the upper bound is changed by the second inequality of Eq. (7). Figure 1 depicts the range of coefficient *λ*_*k*_/2*p*, where *k* is set to the horizontal coordinate, *p* is the vertical coordinate, and the color codes represent the values of *λ*_*k*_/(2*p*). Obviously, |*λ*_*k*_/(2*p*)| ≤ 1, and this equal sign can be achieved in *k* = *N*/2 and *p* = 1. Thus, one can obtain that the AD boundary for *p* = 1 is 1 < *ε* < (1 + *w*^2^)/2, which is consistent with ref.^16^. Furthermore, in the case of local coupling (*p* = 1), the phase diagram can be obtained numerically on the *w* − *ε* plane in Fig. 2. Specifically, we can define an amplitude index *A*_*i*_, and *A*_*i*_ denotes the difference between the global maximum and minimum values of the time series of the *i*th oscillator over a sufficiently long interval. If *A*_*i*_(*i* = 1, …, *N*) turn to zero, AD or OD occurs, otherwise, OS can occur. Moreover, one needs to determine whether the maximum (or minimum) values of all oscillators are the same to distinguish AD and OD, namely, if they are identical, AD appears, otherwise, OD appears. Herein, the green region denotes oscillation death (OD), and purple region is amplitude death (AD), and the white region is limit cycle oscillation (OS). It is worth mentioning that for the OD phenomenon in Fig. 2, it can be divided into two parts by the line *ε* = 1.0, the upper part is the only OD denoted Region I, and the lower part is a coexistence of OD and limit cycle oscillation (OS) called Region II. In addition, here the corresponding theoretical boundaries (red, blue and green lines in Fig. 2) have been described for locally coupled system in ref.^16^.

Through the above analysis, the collective behaviors of the coupled system (Eq. (1)) in the local coupling form (*p* = 1) have been established entirely with the changes of parameters (*ε* and *w*). However, up to now, the evolutions of oscillation patterns remain unclear for the scenario of *p* ≠ 1. For example, does the increasing of coupling range break stability of the AD and OD? How does coupling range affect the transition between AD and OD? And apart from the above dynamic behaviors in the local coupling, what new phenomena can appear for the nonlocal network (1 < *p* < *N*/2)? These questions will constitute the main researches of this paper.

In this work, considering that the transition from local coupling to nonlocal one, we can obtain theoretically the conditions supported AD for different coupling range. However, the theoretical analyses for OD state becomes more difficult due to the appearance of different equilibrium points, which leads to that the researches of OD rely mostly on numerical simulations. Similarly, the changes of OS state also are researched using the numerical methods. Therefore, in the following, for the evolutions of dynamical regimes we will adopt two numerical methods, one is spatiotemporal patterns of coupled system, and the other is the statistical measure–strength of incoherence (SI)^40^. In fact, SI is a suitable statistical method to measure the coherence of the coupled oscillators via using the time series. The utilization of SI will provide a very efficient method to observe the transition processes.

### Effects of coupling range on AD

**Figure 3:**
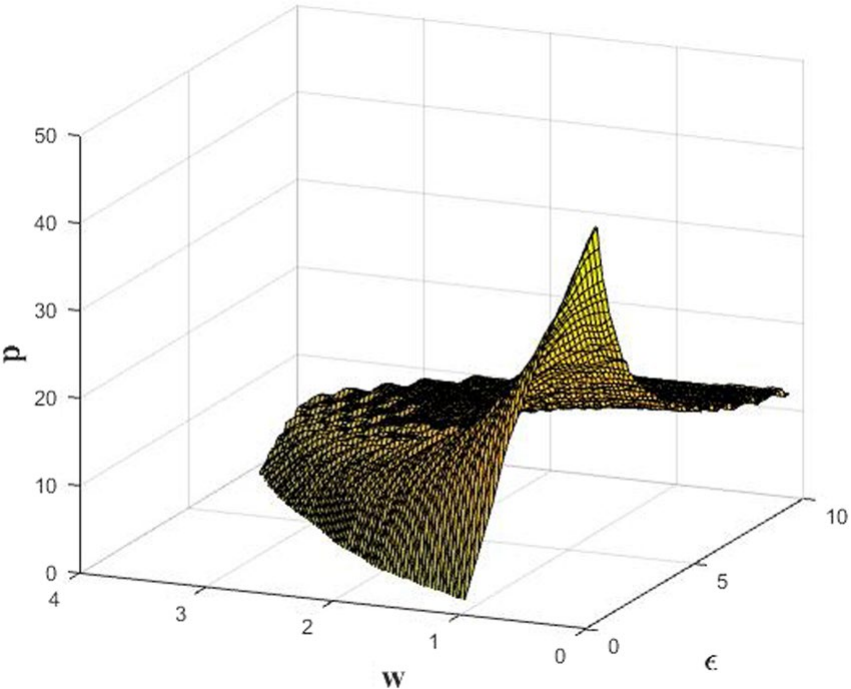
The critical values of *p* in *w* − *ε* plane.

In this subsection, we will investigate the effects of coupling range *p* on the AD state in detail. Firstly, According to the second inequality of Eq. (7), we can derive $|{\lambda }_{k}/2p| < \sqrt{{(\varepsilon -1)}^{2}+{w}^{2}/\varepsilon }$, and we can further obtain numerically the critical values of *p* with the change of *w* and *ε* as shown in the following Fig. 3. Wherein, when *p* exceeds the critical values, Eq. (7) always holds, and AD can occur for any coupling strength (*ε* > 1) and natural frequency (*w* > 0).

**Figure 4:**
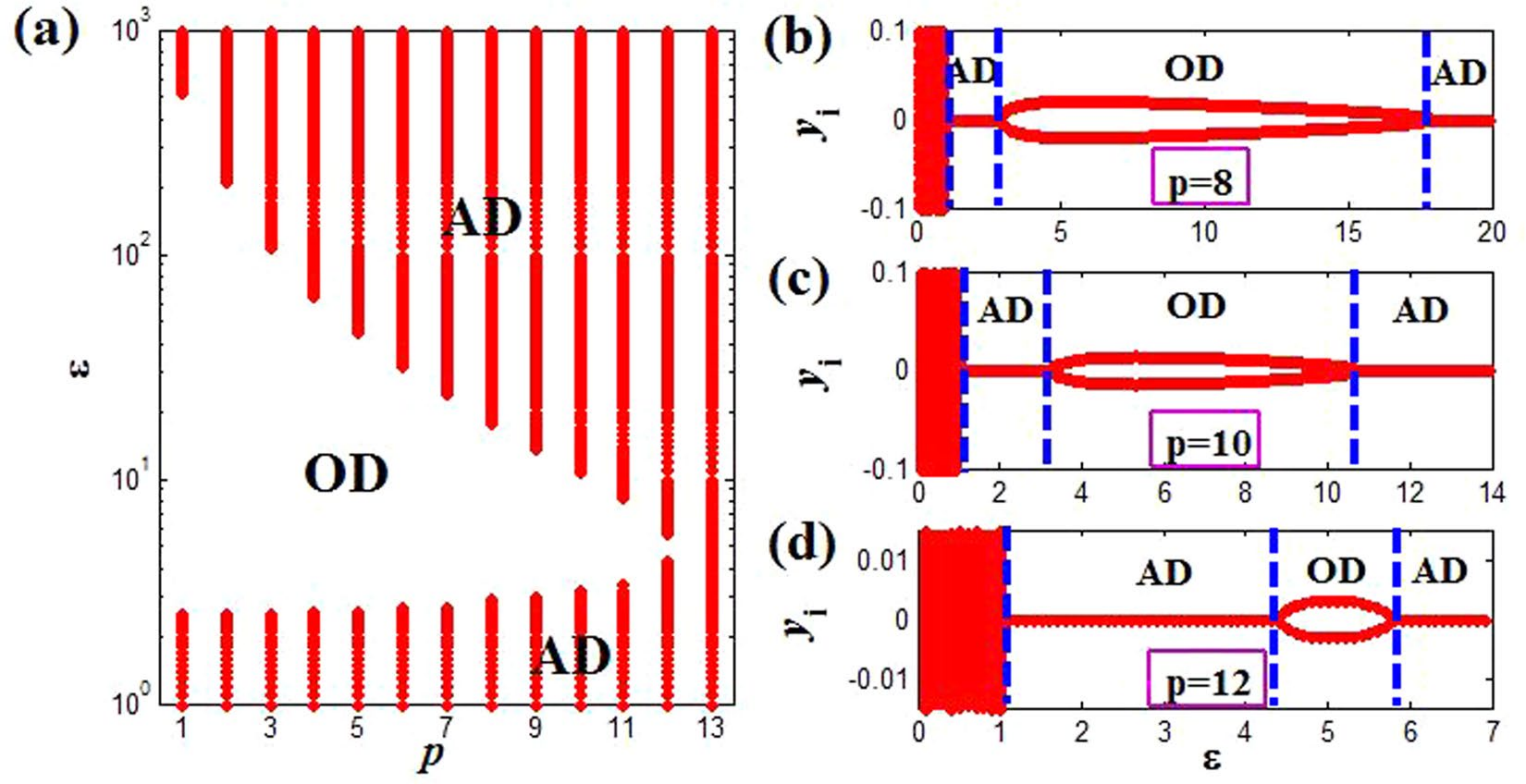
(**a**) The phase diagram of coupled system (Eq. (1)) on the *p* − *ε* plane. The red region corresponds to the AD, and the white region is OD. (**b**–**d**) Bifurcation diagrams for different coupling range *p* = 8, 10 and 12 respectively. It should be illustrated that there are a lot of different stable branches of OD, and we only draw the bifurcation diagram for single oscillator to describe OD state for clarity and simplicity.

In the following, we will show more concretely the range of AD for different *p*. On the one hand, we fix the intrinsic frequency *w* = 2 without loss of generality, the above Eq. (7) can be simplified to

8 $$\,\{\begin{array}{c}\varepsilon  > 1,\\ a{\varepsilon }^{2}-2\varepsilon +5 > 0.\end{array}$$

where *a* = [1 − (*λ*_*k*_/(2*p*))^2^] > 0. According to the properties of quadratic function, one can easily know that the second inequality of Eq. (8) is always correct if Δ = 4 − 20*a* < 0, and it further is equivalent to *p* ≥ 13 through simple algebraic calculations and numerical analyses. That is, as long as *p* ≥ 13 is established, AD will occur for coupling strength *ε* > 1, which agrees with the results in Fig. 3. Besides, for Δ > 0, we also can derive that the ranges of coupling strength ensured AD are $1 < \varepsilon  < (1-\sqrt{1-5a})/a$ and $\varepsilon  > (1+\sqrt{1-5a})/a$ with the change of coupling range *p*. Interestingly, we find numerically that OD state can occur in the interval $(1-\sqrt{1-5a})/a < \varepsilon  < (1+\sqrt{1-5a})/a$, which are shown in Fig. 4(a). Herein, the ranges of AD are gradually expanded, while the OD regions correspond to shrink as *p* increases, and finally only AD state occurs for *p* ≥ 13. Moreover, Fig. 4(b–d) show the bifurcation diagram of the variable *y*_*i*_ (*i* = 1, …, 100) for different coupling ranges *p*. Therein, AD appears for 1 < *ε* < 2.9 and *ε* > 17.9, and OD is in 2.9 < *ε* < 17.9 at *p* = 8. Then AD enlarges to 1 < *ε* < 3.2 and *ε* > 10.8, and OD reduces to 3.2 < *ε* < 10.8 at *p* = 10. Further AD range becomes 1 < *ε* < 4.3 and *ε* > 5.7, and OD narrows to 4.3 < *ε* < 5.7 at *p* = 12. As a result, one can observe clearly that apart from the transition from AD to OD via pitchfork bifurcation, the other transition from OD to AD can also be achieved through inverse pitchfork bifurcation when the natural frequency is fixed. Thus, the changes of coupling range have a significant impact on the AD phenomenon of coupled system for the fixed natural frequency. Namely, the AD state (HSS) is promoted, while the OD state (IHSS) is restrained when the coupling range increases.

**Figure 5:**
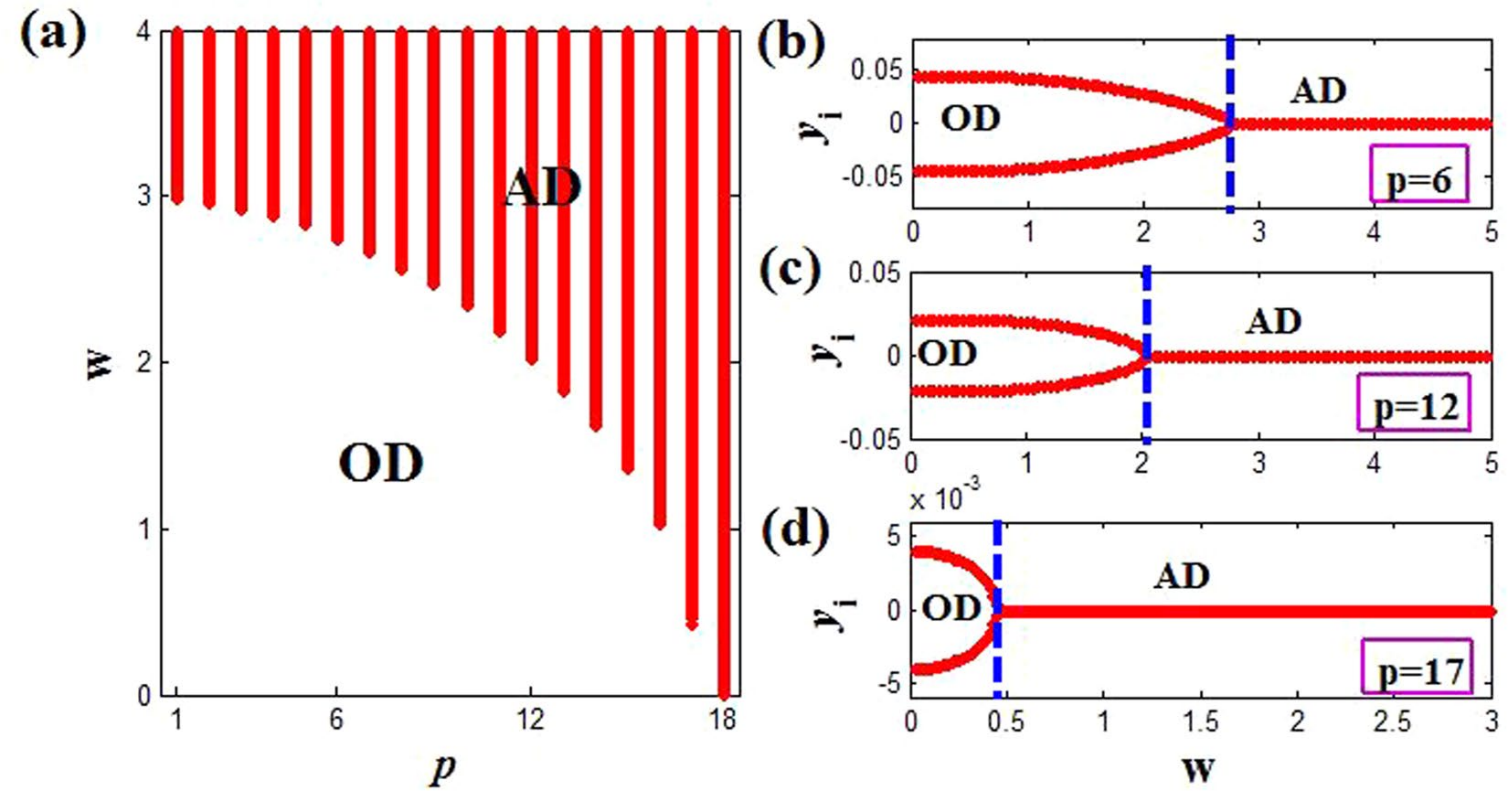
(**a**) The phase diagram of coupled system (Eq. (1)) on the *p* − *w* plane. The red region corresponds to the AD, and the white region is OD. (**b**–**d**) Bifurcation diagrams for different coupling range *p* = 6, 12 and 17 respectively. Here only two OD branches are shown like Fig. 4.

On the other hand, we can also give the coupling strength *ε* = 5.0 to investigate the influence of the coupling range *p* on the natural frequency *w* for inducing AD. Here Eq. (7) can be simplified to

9 $$25[1-{(\frac{{\lambda }_{k}}{2p})}^{2}]-9+{w}^{2} > 0,$$

and it can further be reduced to *w*^2^ > 25(*λ*_*k*_/(2*p*))^2^ − 16, which indicates that the upper bound of *w* to produce AD is infinite, and changing the *p* value only can adjust the lower bound. It can be noticed that if |*λ*_*k*_/(2*p*)| < 4/5 is satisfied (where the threshold of coupling range is *p* = 18), Eq. (9) always holds, and the result also can be obtained directly in Fig. 3. Namely, *w* can become very small for *p* ≥ 18. In addition, we also numerically calculate the minimum natural frequencies for *p* < 18 in Fig. 5(a), where the minimum value of *w* (red region) decreases monotonically with the increasing of *p*. Not only that, the transition from OD to AD through inverse pitchfork bifurcation also can be observed with the increasing of *w*, which can be confirmed by the bifurcation diagram of the variable *y*_*i*_ of coupled system for *p* = 6, 12, and 17 in Fig. 5(b–d). Herein, the critical natural frequencies to achieve the transition are *w* = 2.8, 2.0, and 0.5 respectively. Thus, when the natural frequency varies, we can obtain that the increasing of coupling range can accelerate the transition from OD to AD until OD is eliminated completely.

To end this subsection, we can know that tuning the coupling range, the AD regions can be enlarged remarkably in two directions of both the coupling strength and the natural frequency. Particularly, only AD state can occur for *ε* > 1 on the (*ε*, *w*) plane in Fig. 2 when the coupling range exceeds the threshold. In fact, the different stable fixed points (IHSS) of OD are reduced and ultimately converged into the same steady state (AD) with the increasing of coupling range in the above region, which will be discussed detailedly in next subsection. It is noteworthy that the adjustment of coupling range in the stable regions (AD and OD) makes that the stability of coupled system is not destroyed, and just the number of stable equilibrium points can be changed.

### Effect of coupling range on OD

**Figure 6:**
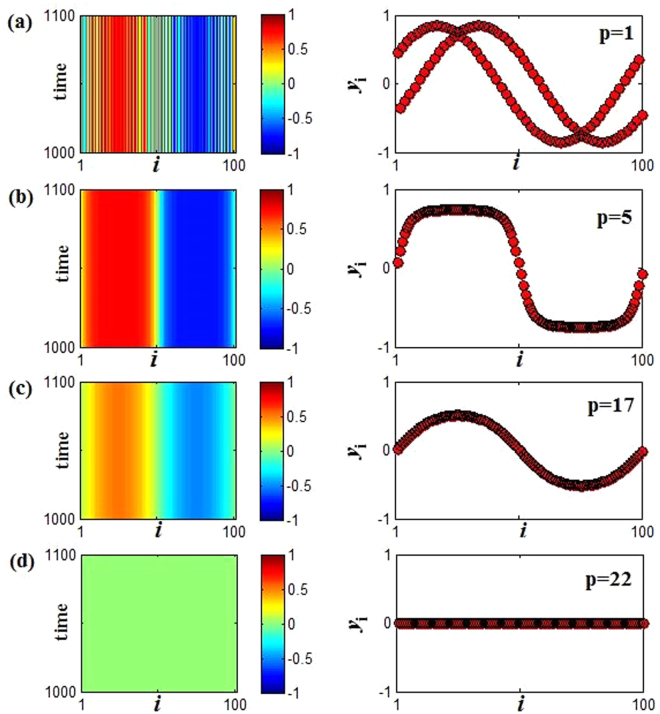
Spatiotemporal patterns (left panel) and the snapshots (right panel) (at *t* = 1000) of the variable *y*_*i*_(*t*) for fixed coupling strength and natural frequency *ε* = 2.0 and *w* = 1.0. (**a**–**d**) The coupling range *p* are changed in turn, *p* = 1, 5, 17 and 22 from top to bottom.

Through the above analyses for AD, it is concluded that OD regime becomes eventually AD as coupling range *p* increases in the Region I. Here we will explore the emerging phenomena in the process of transition from OD to AD. Firstly, we select a point (*w* = 1.0, *ε* = 2.0) in the Region I, further to observe the changes of dynamic behaviors of the coupled system with the increasing of *p* by spatiotemporal patterns and snapshots in Fig. 6. From Fig. 6(a), we can see that the system has numerous stable fixed points for the coupling range *p* = 1, namely different oscillators populate different stable branches (emergence of OD phenomenon). With the increasing of coupling range to *p* = 5, the stable branches of OD reduce, and the neighboring oscillators gradually fall on the same branch to appear the cluster behavior, which is shown in Fig. 6(b). Then, the amplitude of oscillators decreases progressively (see Fig. 6(c)) and vanishes eventually to form AD state when *p* climbs to *p* = 22 (see Fig. 6(d)). Hence, the transition of OD to AD in the Region I can be regarded as two processes: clustering of stability points and decreasing of amplitude. In addition, for the continuous increasing of coupling range *p*, the changes of snapshots are exhibited in the Supplementary Materials (OD1. gif).

For better describing the variations from OD to AD in the Region I, we can use the strength of incoherent measure (SI)^40^ to observe the transition from IHSS (incoherent state) to HSS (coherent state). In fact, the larger is SI the more coherent is the dynamics. The transition from OD to AD is actually a process that the coherence of coupled system is enhanced gradually. We firstly define new variables *ξ*_1,*i*_ = *x*_*i*+1_ − *x*_*i*_ and *ξ*_2,*i*_ = *y*_*i*+1_ − *y*_*i*_, which implies that the neighboring oscillators are coherent for *ξ*_1,*i*_, *ξ*_2,*i*_ → 0 (*ξ*_1,*i*_ = *ξ*_2,*i*_ = 0 for AD state). Then the total number of oscillators is split into *M* bins with equal size *n* = *N*/*M*, and the local standard deviation *σ*_*l*_(*m*) can be derived

10 $${\sigma }_{l}(m)={\langle \sqrt{\frac{1}{n}\sum _{j=n(m-1)+1}^{mn}{[{\xi }_{l,j}-\langle {\xi }_{l}\rangle ]}^{2}}\rangle }_{t},$$

here *m* = 1, …, *M* and $\langle {\xi }_{l}\rangle =(1/N){\sum }_{i=1}^{N}{\xi }_{l,i},l=1,2$. Further the SI is defined

11 $${\rm{SI}}=1-\frac{{\sum }_{m=1}^{M}{s}_{m}}{M},\,{\rm{and}}\,{s}_{m}={\rm{\Theta }}(\delta -{\sigma }_{l}(m)),$$

where Θ is the Heaviside step function, and *δ* is relatively small and represents a certain percentage of difference between maximum and minimum of the variables in the coupled system. We also can note that the smaller *δ* is, the stronger the demand of coherence among the neighboring oscillators is. Here, we take *M* = 10 and *δ* = 0.01, and SI = 0, SI = 1 and 0 < SI < 1 represent AD state, incoherent OD and cluster state respectively. Namely, when the coupled system is in the OD state, all oscillators populate different stable branches, presenting an incoherent state (SI = 1). With the enhancement of coherence, the coupled oscillators can turn to cluster state (0 < SI < 1). Finally, all oscillators are stabilized to the same fixed point to produce AD phenomenon, where the coherence is the strongest (SI = 0), namely, complete coherent state. Therefore, SI can be used to distinguish the transition from OD to AD.

**Figure 7:**
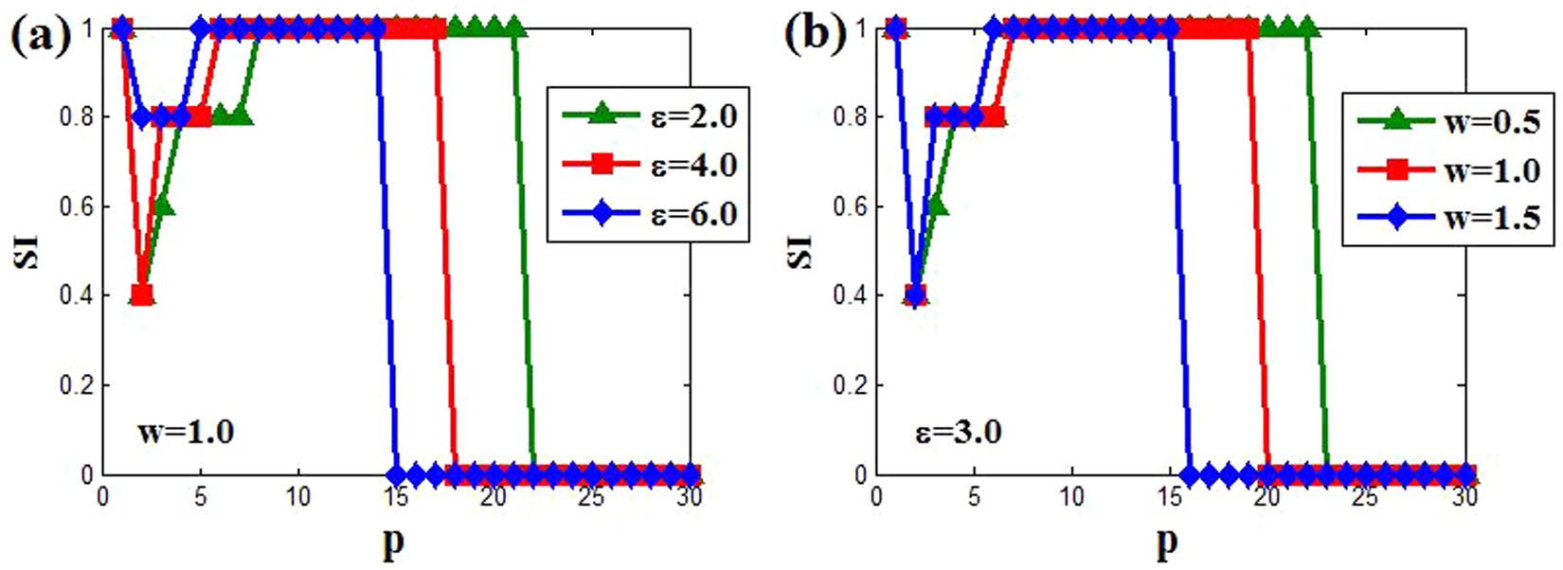
The changes of SI versus to coupling range *p*. (**a**) The natural frequency *w* = 1.0 for different coupling strength *ε* = 2.0 (green triangle), (red square) and *ε* = 6.0 (blue diamond). (**b**) The coupling strength *ε* = 3.0 for different natural frequency (green triangle), *w* = 1.0 (red square) and *w* = 1.5 (blue diamond). Here the parameters *M* = 10 and *δ* = 0.01.

Now, we can draw the change of SI for different points of the Region I with the increasing of *p*. For instance, Fig. 7(a) shows the transition from incoherent OD state to coherent AD via cluster state for different coupling strength *ε* = 2.0,4.0 and 6.0 at fixed natural frequency *w* = 1.0. It can be obtained that the greater the coupling strength *ε*, the smaller coupling range *p* to induced AD is. Similarly, the increasing of natural frequency also can contribute to the transition through Fig. 7(b) for fixed coupling strength *ε* = 3.0. Thus, when the coupling range increases, the transition to AD state gets faster if the coupling strength or natural frequency selects the greater values.

**Figure 8:**
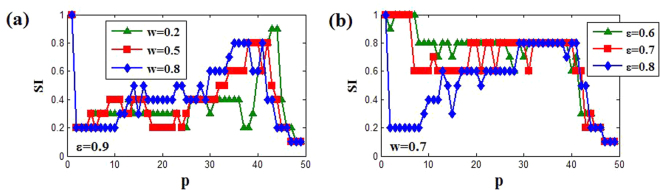
The changes of SI versus to coupling range *p*. (**a**) The coupling strength *ε* = 0.9 for different natural frequency *w* = 0.2 (green triangle), *w* = 0.5 (red square) and *w* = 0.8 (blue diamond). (**b**) The natural frequency *w* = 0.7 for different coupling strength *ε* = 0.6 (green triangle), *ε* = 0.7 (red square) and *ε* = 0.8(blue diamond). Here the parameters *M* = 20 and *δ* = 0.05.

**Figure 9:**
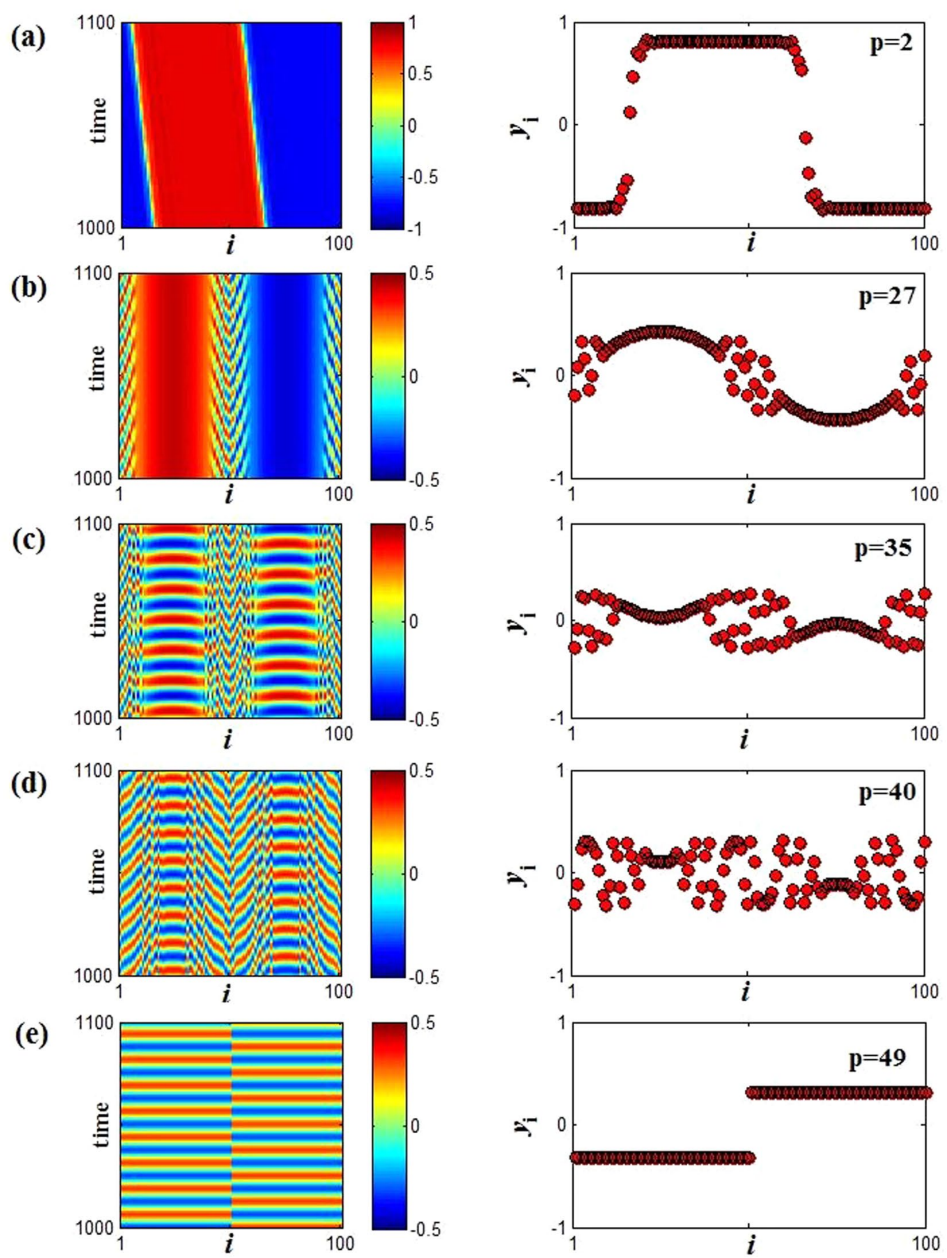
Spatiotemporal patterns (left panel) and the snapshots (right panel) (at *t* = 1000) for the variable *y*_*i*_(*t*) for fixed coupling strength and natural frequency are respectively *ε* = 0.9 and *w* = 0.5. (**a**–**e**) The coupling range *p* are changed in turn, *p* = 2, 3, 27, 45, and 49 from top to bottom.

For the OD state in Region II, when coupling range *p* increases, the stability of coupled system will be destroyed, so finally AD also will not appear. However, we observe that two emerging phenomena, chimera state and anti-synchronization, can arise with the change of coupling range. Similarly, the strength of incoherent measure (SI) also can be utilized to depict the variations of OD state, and the parameters of SI are chosen *M* = 20 and *δ* = 0.05. Owing to the selection of initial condition, the completely coherent state is impossible (SI = 0), and only for SI = 0.1 the coherence reach the strongest (see Fig. 8). So here SI = 0.1, SI = 1 and 0.1 < SI < 1 represent the strongest coherent state, incoherent OD and chimera state respectively. Figure 8 describes the change of SI for the different points in Region II. Wherein, with the increasing of *p*, the oscillation behaviors turn rapidly to chimera state (0.1 < SI < 1) from the incoherent OD state (SI = 1), and finally reaching the coherent state (SI = 0.1). In fact, the smaller SI represents the stronger coherence, which also can be confirmed though spatiotemporal evolutions and snapshots of the variable *y*_*i*_ in Fig. 9 with *ε* = 0.9 and *w* = 0.5(the red square line in Fig. 8(a)). Wherein, for SI = 1 the coherent OD state occurs (see Fig. 6(a) with *p* = 1), and then the coupled system shows traveling wave state when SI = 0.2 (*p* = 2), and SI = 0.4 (*p* = 27), SI = 0.6 (*p* = 35) and SI = 0.8 (*p* = 40) show respectively chimera state behaviors in Fig. 9(b–d). Thus, the coherence between the neighboring oscillators is gradually decreased with the increasing of SI. Until SI = 0.1 (*p* ≥ 47) the oscillators evolve into two synchronization regimes: 1–50 oscillators maintain the same synchronization rhythm, and 51–100 oscillators have another same rhythm synchronous oscillation as shown in Fig. 9(e), which represents the appearance of anti-synchronization. Furthermore, the continuous change of snapshots with *p* is also provided in the Supplementary Materials (OD2. gif).

**Figure 10:**
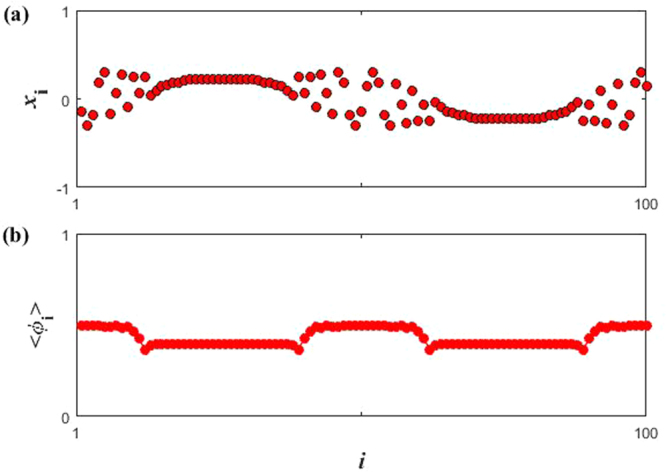
(**a**) The snapshots (at *t* = 1000) for the variable *x*_*i*_(*t*). (**b**) The mean phase velocity profile. Parameter: *ε* = 0.9, *p* = 35 and *w* = 0.5.

Considering that chimera states have various categories, it is important to distinguish the types of chimera states mentioned above. To illustrate this, we calculate the snapshots for the variable *x*_*i*_(*t*) and mean phase velocity $\langle {\varphi }_{i}\rangle$. $\langle {\varphi }_{i}\rangle$ is defined as: ${\langle \varphi \rangle }_{i}={\langle d{\theta }_{i}(t)/dt\rangle }_{{\rm{\Delta }}t}$, and ${\theta }_{i}(t)=\arctan [{y}_{i}(t)/{x}_{i}(t)]$. Here Δ*t* (=1000) denotes the time window over which the average is computed. The mean phase velocity profile is arc shaped to denote the incoherent region of a chimera state, while it is flat to represent the coherent region of the chimera. Take the Fig. 9(e) as example, we can obtain that the chimera behaviors of the coupled system not only with respect to the amplitude, but also the phase through snapshots and mean phase velocity profile in Fig. 10. Besides, we also conducted out the same analysis for the above chimera behaviors and obtained the identical results. Therefore, the chimera states should be called amplitude-mediated chimera^26,27^.

To sum up this subsection, we can obtain that the OD in Region I will convert into AD (reduction of equilibrium points), while the OD in Region II can occur anti-synchronization (destruction of stability of equilibrium points). Consequently, the emergence of two different behaviors is mainly caused by the size of coupling strength, that is, weak coupling strength is conducive to synchronization, whereas strong one contributes to oscillation quenching (AD/OD)^7^.

### Effect of coupling range on LC oscillation

**Figure 11:**
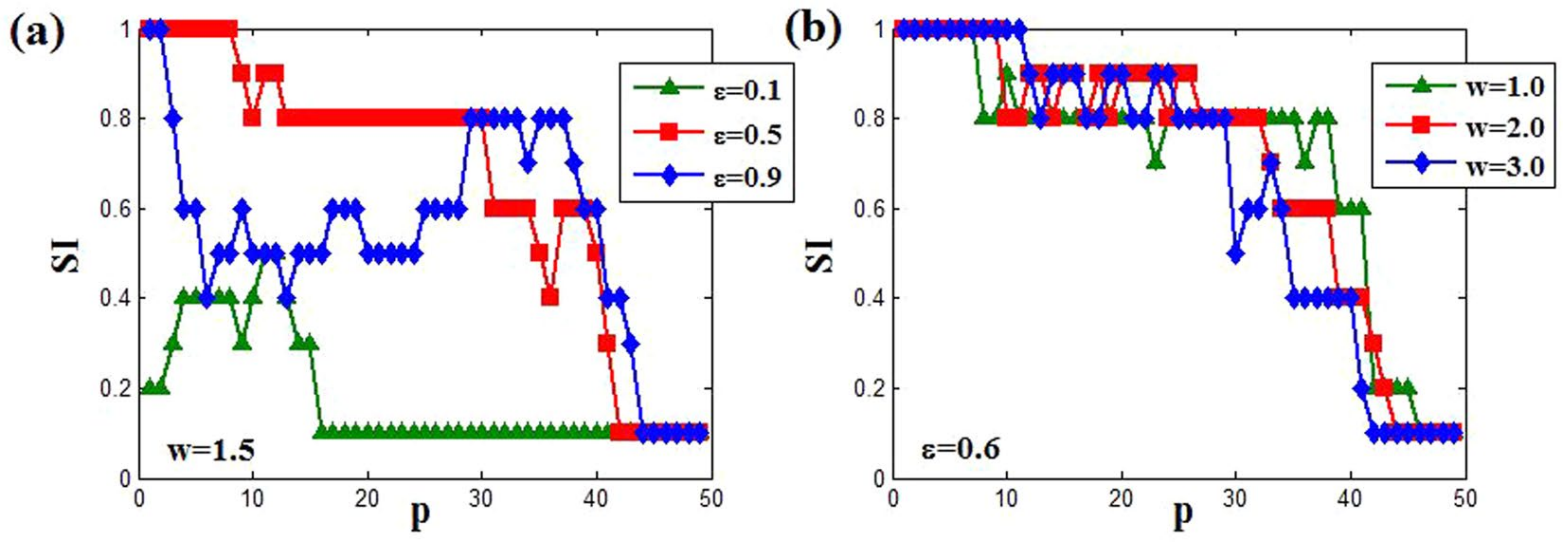
The changes of SI versus to coupling range *p*. (**a**) The natural frequency *w* = 1.5 for different coupling strength *ε* = 0.1 (green triangle), *ε* = 0.5 (red square) and *ε* = 0.9 (blue diamond). (**b**) The coupling strength *ε* = 0.6 for different natural frequency *w* = 1.0 (green triangle), *w* = 2.0(red square) and *w* = 3.0 (blue diamond). Here the parameters *M* = 20 and *δ* = 0.05.

**Figure 12:**
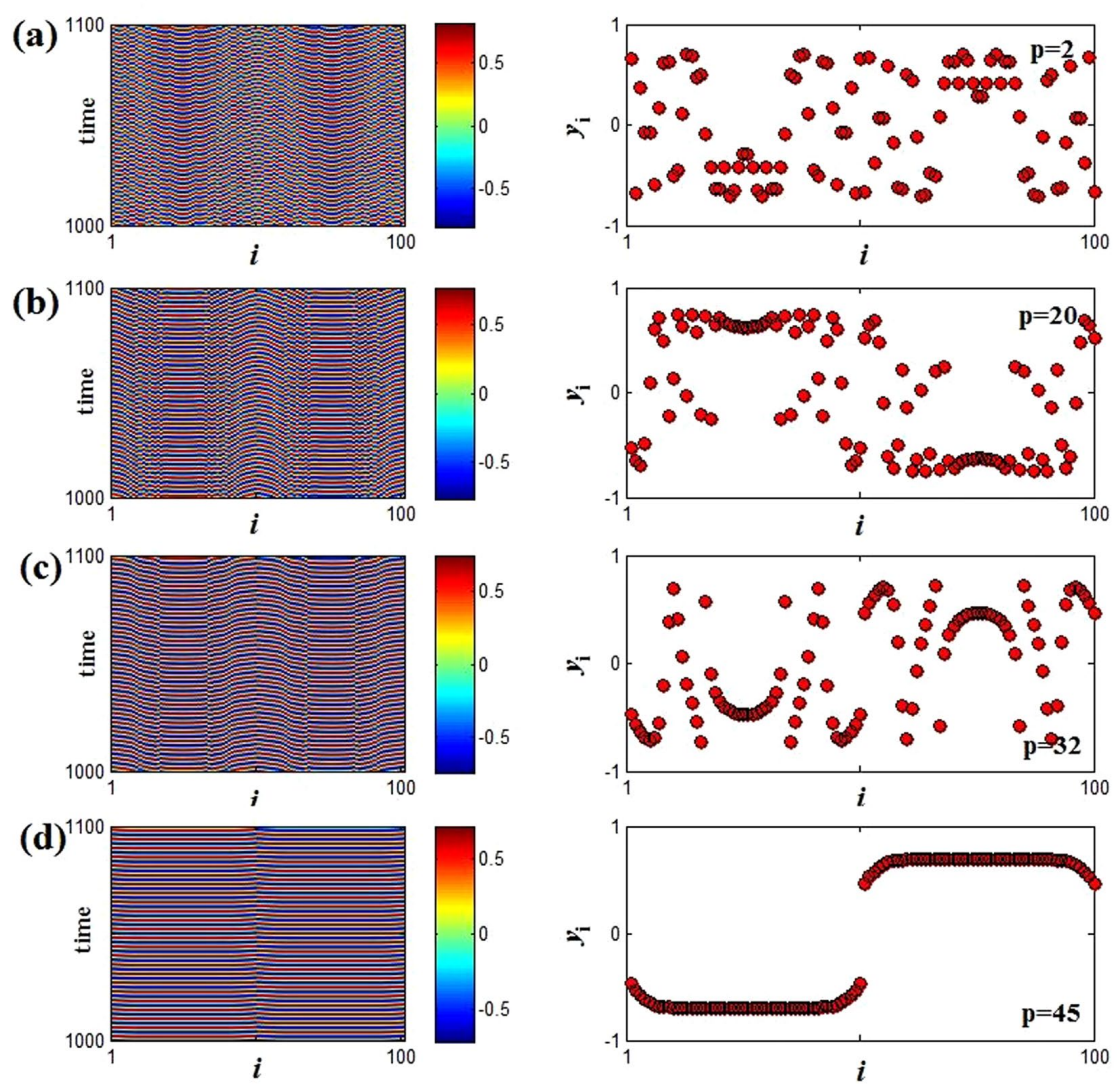
Spatiotemporal patterns (left panel) and the snapshots (right panel) (at *t* = 1000) for the variable *y*_*i*_(*t*) for fixed coupling strength and natural frequency are respectively *ε* = 0.5 and *w* = 1.5. (**a**–**d**) The coupling range *p* are changed in turn, *p* = 2, 20, 32, and 45 from top to bottom.

For the limit cycle oscillation in Fig. 2, we also can use the SI index to describe the transition from incoherent state to coherent state with the change of coupling range. From Fig. 11, it can be clearly observed that the coupled system in incoherent limit cycle oscillation (SI = 1) becomes anti-synchronization state (SI = 0.1) through chimera state (0.1 < SI < 1). However, for the relatively weak coupling strength *ε* = 0.1 (green triangle line in Fig. 11(a)), the transition is achieved directly from chimera state to anti-synchronization state. Moreover, the coupling range induced anti-synchronization (the strongest coherence) is relatively small for smaller coupling strength at fixed natural frequency, while it needs the larger natural frequency at fixed coupling strength. Similarly, we here also describe the spatiotemporal evolutions and snapshots of the variable *y*_*i*_ in Fig. 12 with *ε* = 0.5 and *w* = 1.5 (the red square line in Fig. 11(a)). Wherein, with the decreasing of SI, the coherence of coupled system increases gradually (Fig. 12(a–c)), until finally the anti-synchronization state is reached (SI = 0.1). Therefore, adjusting the coupling range also can achieve the enhancement of coherence among oscillators for OS region. It is worth mentioning that here the observed chimera states are also amplitude-mediated chimera (Fig. 12(b,c)), and the relevant evidences are not shown repeatedly. Besides, the continuous change of snapshots with the increasing of coupling range *p* also is showed in the supplementary materials (LCOS. gif).

## Discussion

To summary, we have researched systematically the collective dynamics behaviors in a ring of *N* nonlocally coupled Stuart-Landau oscillators with conjugate variables. Though increasing the coupling range, the oscillation patterns of nonlinear system can produce notable variations. For relatively strong coupling strength (*ε* > 1), the AD region gradually expands along two directions of both the natural frequency and the coupling strength when the coupling range increases gradually. In fact, only homogeneous steady state (AD) and inhomogeneous steady state (OD) appear for conjugate coupled system in the case of local coupling (*p* = 1). However, with increasing of coupling range, the different stable branches of OD gradually converge to the single equilibrium to generate AD state. Thus, here the increasing of coupling range not only maintains the system’s stability, but also enhances their homogeneity. In contrast, for weak coupling strength (*ε* < 1), as coupling range increases, the stability of OD is destroyed due to the weak interaction, and both OD and OS can convert ultimately into anti-synchronization via amplitude-mediated chimera, which indicates that the coherence of dynamic behaviors of coupled system also is improved. In view of the above fact, it is exclusively illustrated that tuning the coupling range is an effective approach to facilitate coherence/consistency of nonlinear system from incoherence/disordering states.

Finally, through the research of this paper, it is obvious that the increase of coupling range can significantly prompt the incoherent states (OD/OS) to turn to coherent states (AD/anti-synchronization) for conjugate coupled systems, namely the regularity and consistency of the coupled system can be improved. Therefore, we believe firmly that our research will have profound application in experimental realizations and real life. For example, cooperative task of robots, formation flying of UAVs (unmanned aerial vehicle) and the coordinated operation of human organs, all this could be executed to achieve optimal control through finding the proper coupling range.

## Electronic supplementary material

Supplementary dataset 1

Supplementary dataset 2

Supplementary dataset 3
