# Supplementary figures and images for: Enhancing coherence via tuning coupling range in nonlocally coupled Stuart–Landau oscillators
Source: Sci Rep. 2018 Jun 7;8:8721. doi: 10.1038/s41598-018-27020-0 (PMC5992225; doi:10.1038/s41598-018-27020-0)

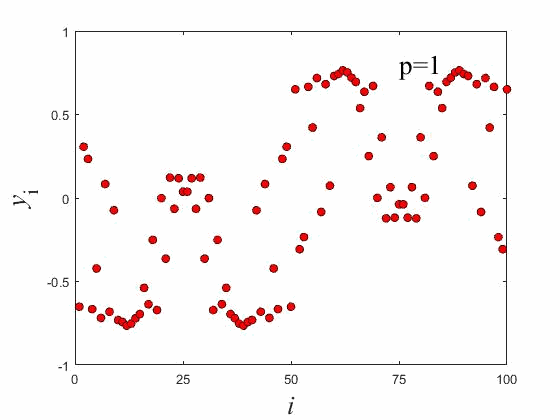

Supplement: Supplementary file 1 — Supplementary dataset 1 [file 41598_2018_27020_MOESM1_ESM.gif]

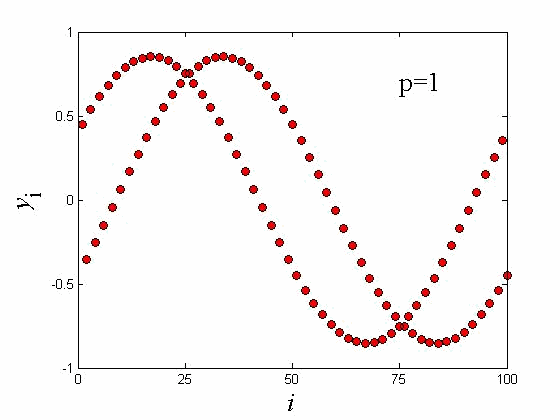

Supplement: Supplementary file 2 — Supplementary dataset 2 [file 41598_2018_27020_MOESM2_ESM.gif]

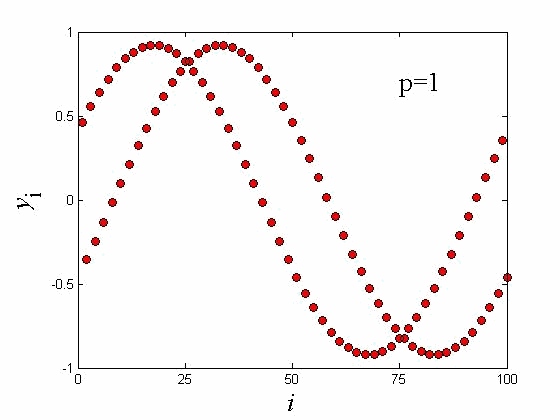

Supplement: Supplementary file 3 — Supplementary dataset 3 [file 41598_2018_27020_MOESM3_ESM.gif]
